# Reduced *Bordetella pertussis*-specific CD4^+^ T-Cell Responses at Older Age

**DOI:** 10.3389/fragi.2021.737870

**Published:** 2022-02-02

**Authors:** Eleonora E. Lambert, Inonge van Twillert, Lisa Beckers, Martien C. M. Poelen, Wanda G. H. Han, Daan K. J. Pieren, Cécile A. C. M. van Els

**Affiliations:** Centre for Infectious Disease Control, National Institute for Public Health and the Environment (RIVM), Bilthoven, Netherlands

**Keywords:** *Bordetella pertussis*, IFNγ ELISpot, CD4^+^ T-cell cytokines, proliferation, infection, aging

## Abstract

Pertussis, a human-specific respiratory infectious disease caused by the Gram-negative bacterium *Bordetella pertussis* (Bp), remains endemic with epidemic years despite high vaccination coverage. Whereas pertussis vaccines and natural infection with Bp confer immune protection, the duration of protection varies and is not lifelong. Recent evidence indicates a considerable underestimation of the pertussis burden among older adults. Whereas the impact of increasing age on Bp-specific humoral immunity has been demonstrated, little is known on immunosenescence of CD4^+^ T-cell responses in the context of Bp. Here, we aimed to address whether increasing age impacts responsiveness of the Bp-specific CD4^+^ T-cells in the memory pool following a clinically symptomatic pertussis infection in whole cell vaccine-primed pediatric and adult cases. Cytokine and proliferative responses and phenotypical profiles of CD4^+^ T cells specific for Bp antigens at an early and late convalescent timepoint were compared. Responses of various Th cytokines, including IFNγ, were significantly lower in older adults at early and late timepoints post diagnosis. In addition, we found lower frequencies of Bp-specific proliferated CD4^+^ T cells in older adults, in the absence of differences in replication profile. Phenotyping of Bp-specific CD4^+^ T cells suggested reduced expression of activation markers rather than increased expression of co-inhibitory markers. Altogether, our findings show that the magnitude and functionality of the Bp-specific memory CD4^+^ T-cell pool decrease at older age. Declined CD4^+^ T-cell responsiveness to Bp is suggested to contribute to the burden of pertussis in older adults.

## Introduction

Pertussis is an acute and severe disease of the respiratory tract and is caused by the highly infectious and human-specific Gram-negative coccobacillus *Bordetella pertussis* (Bp). Pertussis can cause serious illness in people of all ages (Hewlett and Edwards, 2005) and can even lead to fatality in newborns and young infants without protective (maternal) antibody levels (Paddock et al., 2008)*.* It is a vaccine preventable disease, but despite high vaccine coverage, it remains endemic with outbreaks every three to 5 years (van der Maas et al., 2013; Tan et al., 2015). For a long time, pertussis was considered to be a childhood disease (Gordon and Hood, 1951), but substantial evidence has been put forward that it also affects adolescents, adults and elderly (reviewed in (Rothstein and Edwards, 2005; Kandeil et al., 2019)). The true number of pertussis cases in older age groups are likely underestimated due to under identification (McGirr et al., 2013) by missed diagnoses or no medical visit, as severity of symptoms is lower compared to infants. A large burden of disease may therefore occur in older (vulnerable) adults and elderly, facilitating ongoing Bp transmission.

Pioneering studies in mice have shown that especially CD4^+^ T cells producing IFNγ are imperative for controlling Bp bacterial load (Mills et al., 1993; Barbic et al., 1997; Mahon et al., 1997; Leef et al., 2000) and reviewed in (Lambert et al., 2019). Moreover, transferred CD4^+^ T cells but not CD8^+^ T cells were shown to clear Bp infection in convalescent mice (Mills et al., 1993). Neither natural infection, nor vaccination provide life-long immunity to protect against pertussis (Wearing and Rohani, 2009), however epidemiological studies indicate more durable responses induced by natural infection and whole cell pertussis vaccination as compared to acellular pertussis vaccination (Klein et al., 2012; Sheridan et al., 2012; Klein et al., 2013). More sustained protection is associated with skewed CD4^+^ T-cell programming: natural infection and priming with whole cell pertussis vaccination induce programming towards T helper (Th1) and Th17 CD4^+^ T cells, as opposed to Th2/Th17 skewed CD4^+^ T-cell memory responses induced by acellular pertussis vaccination (Ryan et al., 1998; Esposito et al., 2001; Mascart et al., 2003; Vermeulen et al., 2010; Warfel et al., 2014).

Whereas waning immunity has been suggested to explain the occurrence of pertussis in all age groups (reviewed in (van Twillert et al., 2015)), especially aging of the immune system or ‘immunosenescence’ (Gruver et al., 2007) may be a major factor that contributes to pertussis incidence amongst older adults. Immunosenescence of the T-cell population has been widely studied and can result in dysfunctional T-cell responses, which in turn may contribute to increased susceptibility to infection and poor vaccination responses (Akbar et al., 2016). Within the global T-cell population, the total number of T cells declines, memory T cells accumulate, and the naïve T-cell pool reduces (reviewed in (Chinn et al., 2012; Dixit, 2012)), which negatively affects the response to new pathogenic encounters. Moreover, in mice and in humans, immunosenescence of CD4^+^ and CD8^+^ T cells has been associated with a rise of co-inhibitory marker expression, such as PD-1 (Lages et al., 2010), CTLA-4 (Leng et al., 2002), TIGIT (Song et al., 2018), and the transcription factor Helios (Pieren et al., 2021). Currently, Bp-specific immune responses in the population are mostly memory-based, in view of high vaccination coverage during many decades, as well as endemic and epidemic circulation of Bp. It is not known, however, whether antigen-specific CD4^+^ T-cell memory to Bp is subject to aging. To advance our understanding of age-related differences in Bp-specific CD4^+^ T-cell responsiveness, we compared cytokine profiles, proliferative capacity and phenotype of Bp-specific CD4^+^ T-cell populations detectable in time after a clinically symptomatic Bp infection in children, adults and older adult participants in a unique cohort of ex-pertussis cases, sharing a history of primary whole cell pertussis vaccination during infancy.

## Material and Methods

### Ethics Statement

Participants were included from two clinical studies. The first clinical study comprised a cross-sectional observational study in Dutch symptomatic pertussis cases (Specifieke Kinkhoest Immuniteit; SKI; 2008–2012) (Han et al., 2013; van Twillert et al., 2014). This study was approved by the accredited Review Board STEG, followed by management of the METC UMC Utrecht (CCMO nr: NL16334.040.07). The second clinical study was a longitudinal observational study in Dutch symptomatic pertussis cases (Immfact; 2014–2020). This study was approved by the accredited Medical Research Ethics Committee “METC Noord-Holland” (Alkmaar, the Netherlands) followed by management of the METC MEC-U (Utrecht, the Netherlands) (CCMO nr: NL4679.094.13). All participants provided written informed consent for the collection of samples, the usage of a completed questionnaire regarding clinical symptoms and vaccination history, and the subsequent immunological analyses. Informed written consent for minor participants was provided by both parents or guardians of participants. These studies were conducted in compliance with the principles of the Declaration of Helsinki.

### Study Population

Participants from the SKI study consisted of 58 clinically symptomatic (ex) pertussis patients who donated blood at a single known, either early or late, time point after their laboratory confirmed diagnosis. For analysis, subjects were classified into a young group (median age of 12 years; range 11–15 years, n = 21), referred to as youngsters (Y), and an adult group referred to as (A) (median age of 38 years; range 25–56 years, n = 37). Youngsters and adults were subcategorized according to the time elapsed between their date of clinical pertussis diagnosis and date of blood sampling (τ, in months), indicated as sampled in *early phase* (τ1 < 4 months, or in *late phase* τ2 ≥ 8 months, no upper limit for inclusion). Late phase cases were excluded on serological criteria, if having an IgG plasma level specific for Pertussis Toxin (PT) ≥ 62.5 IU/ml, indicative for an additional non-diagnosed exposure (de Melker et al., 2000; de Greeff et al., 2010). Participants of the Immfact study consisted of 16 clinically symptomatic pertussis patients who donated blood at two longitudinal time points after their laboratory confirmed diagnosis. Here, youngsters (Y) with a whole cell priming background (median age of 16 years, range 12–23 years, n = 9) and older adults (O) (median age of 71 years, range 60–78, n = 7) were sampled at τ1 ≤ 3 months post diagnosis (*early phase*) as well as at 8 ≤ τ2 ≤ 12 months post diagnosis (*late phase*). For details of cohorts, see Supplementary Figure S1. All participants from both clinical cohorts and of all age groups were primed during their first year of life and according to their birth cohort with multiple doses of the Dutch whole cell vaccine (in use until the year 2005 when the Dutch National Immunization Programme switched to an acellular primary pertussis vaccine for infants). As by their birth cohort and the Dutch national immunization programme, participants did not receive any pertussis booster dose(s), except for three younger participants from the Immfact cohort. At the age of 4-years these participants received an acellular pertussis booster dose at school entrance, which was programmatically introduced in the Netherlands in 2001. The Dutch National Immunization Programme does not include further adolescent or adult acellular pertussis booster doses. Maternal pertussis immunization was only introduced in December 2019.

### Pertussis Protein Antigens and Peptide Pools

P.69 pertactin (PRN) was recombinantly expressed and purified from *E. coli* as described previously (Hijnen et al., 2005). PT and Filamentous Hemagglutinin (FHA) were both obtained from Kaketsuken, Japan. Presence of *E. coli* LPS or *B. pertussis* LOS was ruled out (<0.015 EU/ml) based on Limulus Amebocyte Lysate (LAL) testing. Pools of synthetic immunogenic Bp peptides were purchased; Bp132, a peptide pool consisting of 132 immunogenic peptides derived from PT, FHA, PRN and Fimbriae 2/3 (Bancroft et al., 2016) (Pepscan); PT peptide pool, consisting of 18-mers spanning the PT S1 subunit with 12 amino acids overlap (in-house synthesis, P. Hoogerhout); FHA peptide pool and PRN peptide pool, consisting of only the FHA or PRN-specific peptides from the Bp132 peptide pool, respectively (Pepscan).

### Blood Sampling and PBMC Isolation

Venous blood samples were collected in the SKI study using CPT Mononuclear cell preparation tubes (BD) and in the Immfact study using vacutainer blood collection tubes (BD). Peripheral blood mononuclear cells (PBMCs) were isolated using standard procedures and frozen in 10% DMSO. Samples were kept at −80°C overnight and then stored at −135°C.

### *In vitro* T-Cell Re-Stimulation

PBMCs were quickly thawed at 37°C and *in vitro* cultured as described elsewhere (Schure et al., 2012) with minor modifications. Briefly, SKI PBMC samples were cultured for 5 days at 37°C, 5% CO_2_, in 96-well U-bottom plates at 3.0 × 10^5^ viable cells in 150 µL/well in replicate wells per condition in AIM-V medium (Gibco, Invitrogen, United States) containing 5% human AB serum (Harlan, United Kingdom) (AIM-V+) only (negative control), or in the presence of 5 μg/ml of PT (heat-inactivated), FHA or PRN, or of 5 μg/ml of pokeweed mitogen (Sigma Chemicals, United States) (positive control). Alternatively, Immfact PBMC samples were labelled with CellTrace Violet (Thermofisher) at a final concentration of 0.5 µM and cultured for 6 days at 37°C, 5% CO_2_, in loosely capped 5 ml tubes (Falcon, BD) at 2.0 × 10^6^ in 1 ml/tube in RPMI 1640 medium (Gibco, Invitrogen, United States) containing 5% human AB serum (Harlan, United Kingdom; RPMI+) in medium only (negative control) or in the presence of PT S1 peptide pool, Bp132, PRN peptide pool, FHA peptide pool (all at 0.1 µM per individual peptide) or anti-CD3 and CD28 antibodies (at 0.5 μg/ml and 1 μg/ml, respectively, positive control). For both SKI and Immfact samples aliquots of culture supernatants were collected on day 5 and stored at −80°C for subsequent quantification of cytokine levels. After culture, SKI and Immfact PBMCs were harvested on day 5 and day 6, respectively, for further analyses. Depending on the amount of PBMCs available per sample, one or more *B. pertussis* antigenic stimulations were tested apart from the negative control.

### IFNγ ELISpot Assay

 5-stimulated PBMC (SKI) samples were spun down and after collection of supernatants, cells were reconstituted in culture medium and transferred to anti-human IFNγ (Mabtech, Sweden) coated ELISpot plates (Millipore, United States) at a starting concentration of 1.4 × 10^5^ cells in 150 µL/well and three times two-fold serially diluted. Plates were incubated for approximately 20 h at 37°C, 5% CO_2_, then after four washing steps and one cell-lysing step, incubated with 1 μg/ml anti-human IFNγ (Mabtech), followed by peroxidase labeled extravidin (Sigma) and BCIP/NBT (KPL, United States). After development, the plates were air-dried at room temperature prior to analysis of numbers of IFNγ spot forming cells (SFC), assessed by an automatic computer-assisted ImmunoScan-Pro reader (CTL Europe, Germany). Results are expressed as IFNγ SFC/100.000 PBMCs with a lower detection limit of 0.1 IFNγ SFC/100.000 PBMCs.

### Flow Cytometry

CellTrace-labelled day 0 and day 6-stimulated Immfact PBMCs were washed and labelled at 4°C for cell surface and intracellular markers with the following antibodies: anti-CD3-Alexa700 (clone SK7), anti-CD4-BV711 (clone OKT-04), anti-CD8-BV785 (clone RPA-T8), anti-CD45RO-PerCP-Cy5.5 (clone UCHL1), anti-FoxP3-Alexa647 (clone 259D), anti-PD-1-BV605 (clone EH12.2H7), anti-CD127-BV650 (clone A019D5), anti-CTLA-4-PE (clone L3D10), anti-TIGIT-PE/efluor-610 (clone MBSA43), anti-Helios-PE-Cy7 (clone 22F6), anti-CD27-BUV395 (clone L128), anti-CD25-BUV737 (clone 2A3) and Live-dead ZOMBIE NEAR IR-APC-Cy7. Acquisition was performed on a BD LSR Fortessa X-20 (BD Biosciences, Franklin Lakes, NJ, United States). Subsets of CD4^+^ T cells were identified based on combined marker expression on gated CD3^+^CD4^+^ cells. Frequencies and phenotypes of proliferating antigen-specific CD4^+^ T cells were determined by analyzing sequential halving of the CellTrace Violet fluorescence intensity combined with performing surface phenotypic and intracellular marker labelling. Percentages of proliferated CD4^+^ T cells found after medium stimulation conditions were subtracted from percentages of proliferated CD4^+^ T cells after Bp-antigen stimulation, when comparing proliferative CD4^+^ T-cell responses between groups. For both replication index calculations (by proliferation modeling) as well as gating analyses, FlowJo software was used (Tree Star, Ashland, OR, United States).

### Cytokine Multiplex Bead-Based Immunoassay

Concentrations of the cytokines IL-2, IFNγ, TNF-α, IL-5, IL-13, IL-17A and IL-10 in day 5 culture supernatants were determined in pg/mL according to manufacturer’s instructions using a commercial multiplex bead-based immunoassay kit (Bio-Rad, United States) (SKI samples) or using the LEGENDplex Human Th cytokines kit (BioLegend) (Immfact samples), and expressed in [pg/ml]. Measurements and data analysis were performed with a Bio-Plex 200, using Bio-Plex Manager software or flow cytometry (FACS Canto II), respectively.

### Dimensionality reduced analyses

Dimensionality-reduced analyses (viSNE) of flow cytometry data were performed in Cytobank (www.Cytobank.org). Prior to the viSNE plots, CD4^+^ proliferating events were exported and pooled per culture condition, age group and timepoint. The number of cells included in the analysis was 2.3 × 10^5^ and was equal for youngsters and older adults.

### Statistical Analysis

For data analysis and visualization of data, GraphPad Prism (GraphPad Software version 8.4.1) was used. Statistical significance of differences was analyzed with the nonparametric Mann-Whitney *t*-test, when comparing stimulated and unstimulated conditions of samples, and the Wilcoxon matched pairs signed-rank test, when comparing longitudinal samples from individuals. For all analyses, *p* values <0.05 were considered statistically significant.

## Results

### Adults Show Lower Frequencies of PT-Specific IFNγ-Producing Cells and IFNγ Levels in the Early Phase After Clinical Pertussis

**FIGURE 1:**
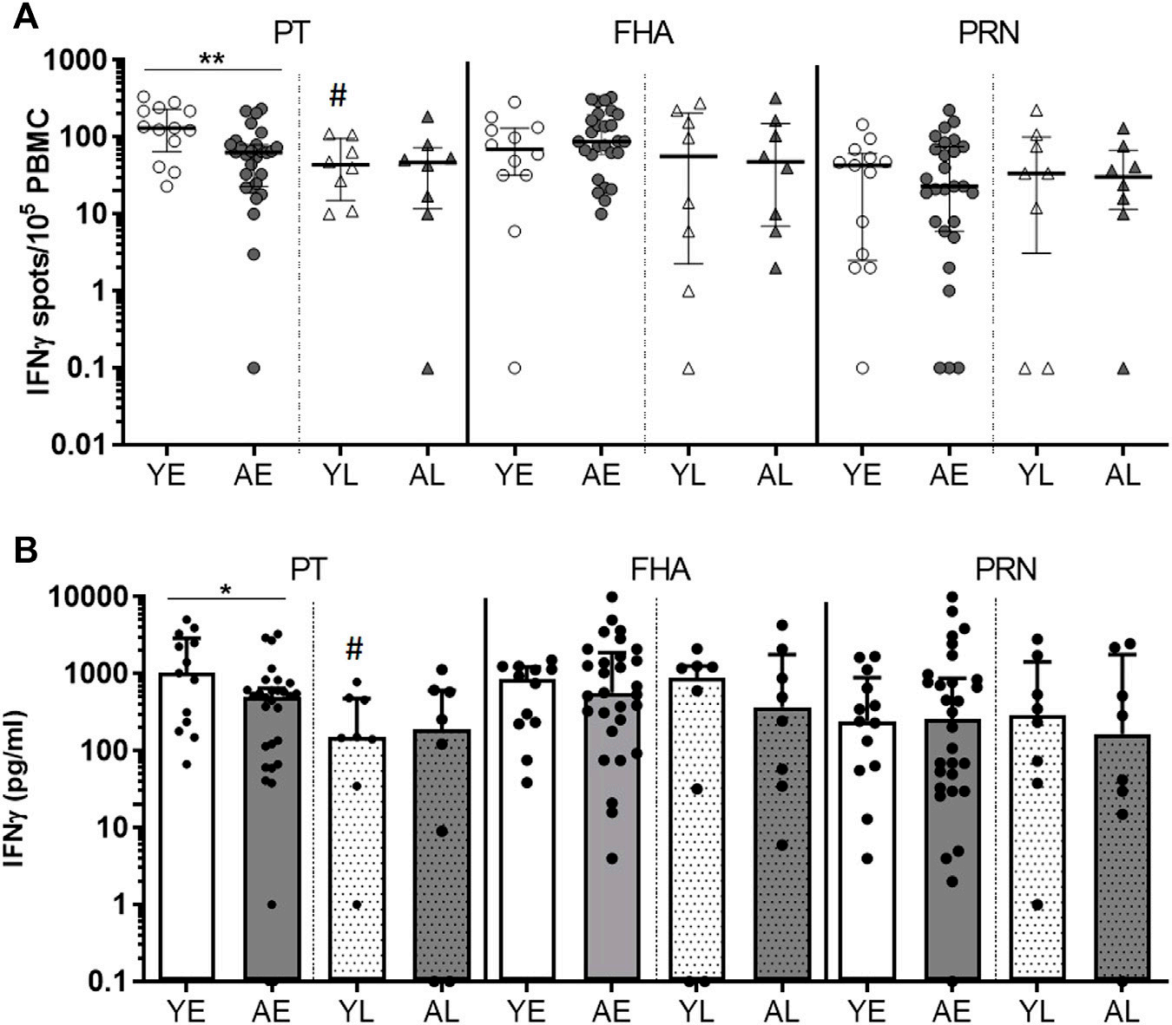
Adults show lower frequencies of PT-specific IFNγ-producing cells and IFNγ levels in the early phase after clinical pertussis (SKI). PBMCs of youngsters (“Y”) and adults (“A”), from an early timepoint (‘E’; within 4 months after diagnosis), and late timepoint (“L”, 8–80 months) after clinical infection were stimulated with Bp proteins PT **(left panels)**, FHA **(middle panels)** and PRN **(right panels)** for 5 days. **(A)** Numbers of IFNγ-spot forming cells per 10^5^ PBMCs measured by ELISpot. **(B)** IFNγ levels in culture supernatants by Luminex and expressed as [pg/ml]. Symbols show individual cases, while lines and bars indicate medians and interquartile ranges. Statistical significance was calculated with Mann Whitney *U*-test. *p < 0.05, **p < 0.01; # = significantly lower compared to the early phase.

Production of IFNγ is an important parameter for an effective immune response to a Bp infection (Mills et al., 1993; Ryan et al., 1997; Hafler and Pohl-Koppe, 1998). We first set out to explore IFNγ production in total PBMC cultures stimulated with Bp protein antigens to compare these responses between youngsters and adults. To determine the impact of age on functionality of Bp-specific responses, we studied IFNγ responses in early and late phase post-infection PBMC samples from youngsters and adult pertussis patients of the SKI cohort with a similar whole cell pertussis vaccine priming background. Production of IFNγ of 5-day cultured PBMCs stimulated with protein antigens Pertussis Toxin (PT), filamentous hemagglutinin (FHA) and pertactin (PRN) were determined by ELISpot. Geomean number of IFNγ SFC ranged between 11–118 per 1 × 10^5^ PBMCs, depending on the stimulation, age and timepoint. The frequency of IFNγ-producing cells in response to PT was significantly lower in adults compared to youngsters in the early phase (Figure 1A). Waning of the PT response from the early to late phase was observed in youngsters but not in adults. In the early phase response to FHA, adults showed comparable median frequencies of IFNγ-producing cells (Figure 1A), which did not decline over time. In response to PRN, no age-related differences in numbers or patterns over time were found for IFNγ-producing cells (Figure 1A). Similar to findings on the number of IFNγ-producing cells, the level of IFNγ measured in culture supernatants was significantly lower in PT-stimulated samples of adults compared to youngsters in the early phase of the response (Figure 1B). In summary, these data suggest age-related differences in IFNγ responses to PT in the early phase after clinical pertussis infection.

### Adults Show Lower Levels of Secreted Th-type Cytokines After Pertussis Infection

**FIGURE 2:**
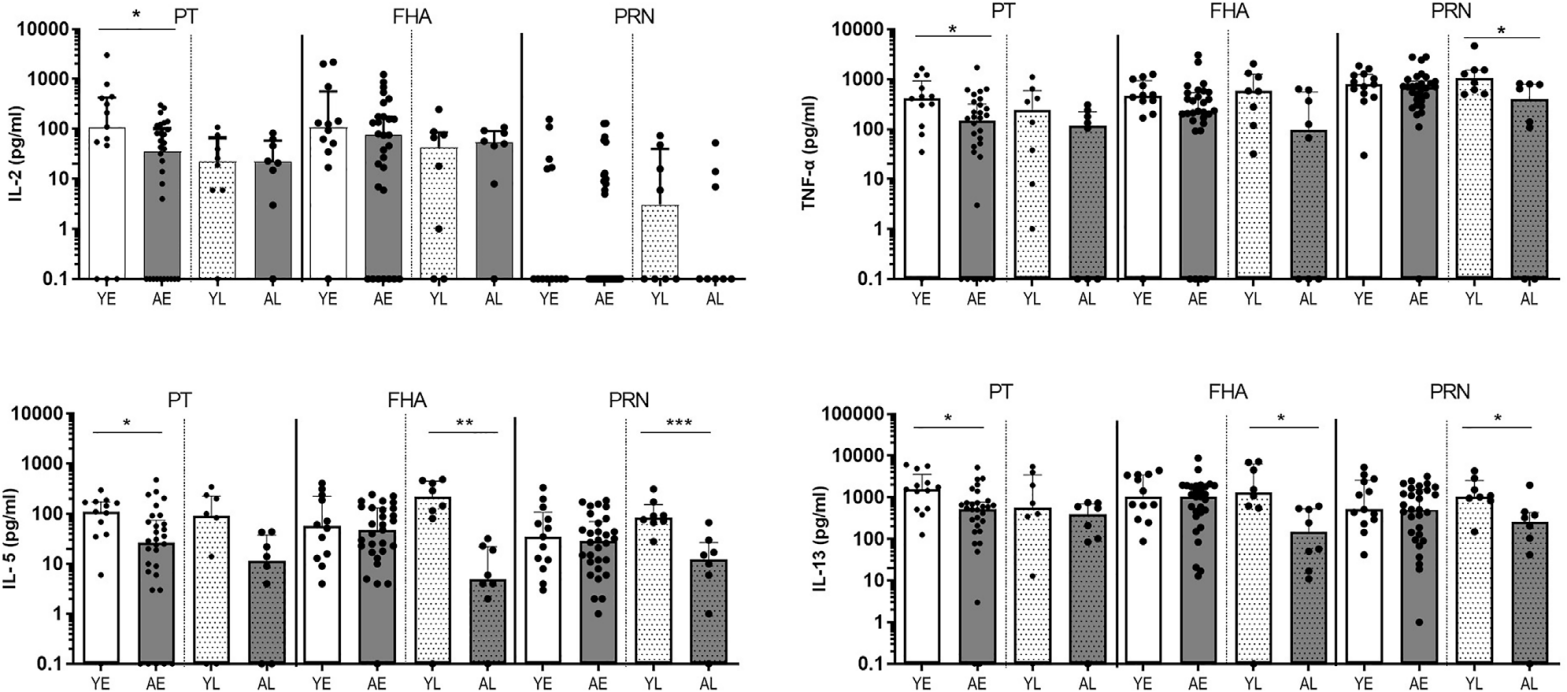
Adults show lower levels of secreted Th-type cytokines after pertussis infection (SKI). Bar graphs show the concentration of IL-2, TNF-α, IL-5, and IL-13 in supernatants of PBMCs *in vitro* stimulated with Bp proteins PT **(left panels)**, FHA **(middle panels)** and PRN **(right panels)** in youngsters (“Y”, white bars) and adults (“A”, dark bars) in early (“E”, open bars) and late (“L”, dotted bars) phase after clinical diagnosis. Dots show individual cases while bars indicate medians and interquartile range. Statistical significance was calculated with Mann Whitney *U*-test. *p < 0.05, **p < 0.01, ***p < 0.001.

Next, we extended our analyses of cytokines produced in total PBMC cultures stimulated with protein antigens in youngsters and adults from the SKI cohort by measuring Th1 (IL-2, TNF-α), Th2 (IL-5, IL-13) (Figure 2), Th17 (IL-17) and regulatory (IL-10) associated cytokines (Supplementary Figure S2). Overall, adults presented with lower levels of cytokines than youngsters in both early and late phase after clinical pertussis infection. We found significantly lower levels of Th1-associated cytokines IL-2 and TNF-α, and Th2-associated cytokines IL-5 and IL-13 in PT-stimulated culture supernatants in adults compared to youngsters in the early phase (Figure 2). In FHA-stimulated samples, significantly lower levels of Th2 associated IL-5 and IL-13 cytokines were found in the late phase in adults compared to youngsters (Figure 2). In addition, PRN-stimulated culture supernatants had significantly lower levels of IL-5, IL-13 and TNF-α in the late phase. Levels of IL-17A and IL-10 were comparable between the two age groups (Supplementary Figure S2). Together, these data indicate that in various phases after clinical pertussis infection, Bp specific cytokine responses, typically mediated by CD4^+^ T cells, are reduced at older age.

**FIGURE 3:**
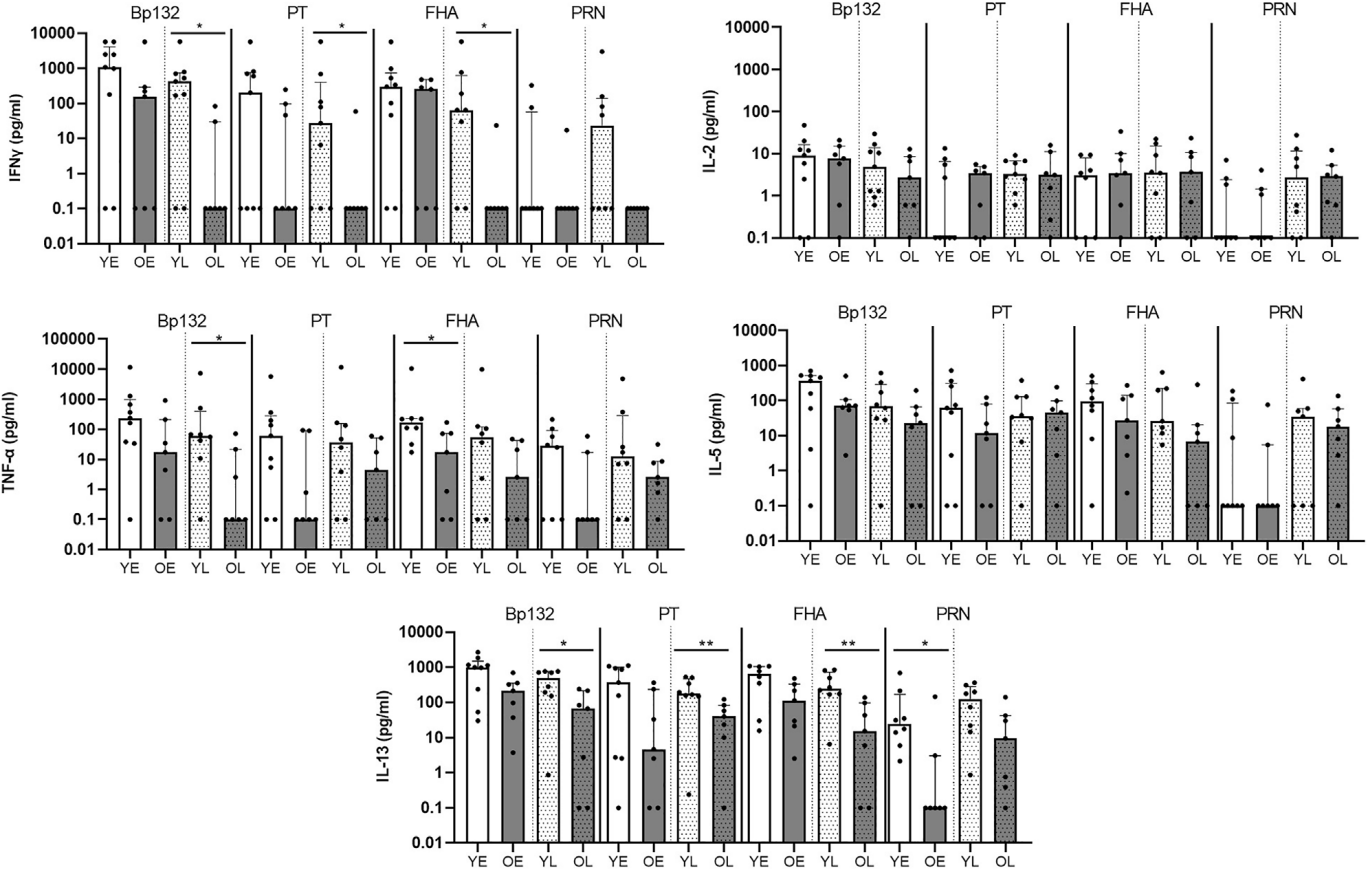
Longitudinal follow up of older adults confirms lower cytokine production after Bp infection (Immfact). Bar graphs show the concentrations of IL-2, TNF-α, IL-5, and IL-13 in supernatants of PBMCs *in vitro* stimulated with Bp peptide pools Bp132, PT S1 (shown as PT), FHA and PRN, as indicated, in youngsters (“Y”, white bars) and older adults (“O”, dark bars) in early (“E”, open bars) and late (“L”, dotted bars) phase after clinical diagnosis. Dots show individual cases while bars indicate medians and interquartile range. Statistical significance was calculated with Mann Whitney *U*-test. *p < 0.05.

Longitudinal follow up confirms lower levels of Th-type cytokines after Bp infection in older adults The lower Bp-antigen specific cytokine responses found in the older age group suggested that either the proportion of Bp-antigen specific functional memory CD4^+^ T cells responding in this group was smaller, or its proliferative capacity was impaired compared to youngsters. Immunosenescence of CD4^+^ T-cell proliferation has been described at increased age (Jiang et al., 2007). To shed more light on the mechanism, we designed a flow-based assay, allowing the analysis of Bp antigen-specific proliferation of CD4^+^ T cells, as well as exploring several cellular markers linked to T-cell immunosenescence. Here, we selected paired samples obtained at fixed time points in the early and late phase post clinical pertussis from whole cell vaccine primed youngsters and older adults in the Immfact study (Supplementary Figure S1). In contrast to stimulations with whole protein antigen, we now used peptide pools of separate and combined Bp antigens of which the epitopes have previously been described as immunogenic and optimal for binding to MHC class II (Bancroft et al., 2016) and therefore allowed us to assess the response of antigen-specific CD4^+^ T cells. First, we compared cytokine production in 5 days culture supernatants of PBMCs from youngsters and older adults post clinical pertussis infection longitudinally (Figure 3, Supplementary Figure S3). In older adults, IFNγ and IL-13 levels were significantly lower in response to peptide pools Bp132, PT S1 and FHA in the late phase. In addition, lower levels of TNF-α were detected in response to Bp132 (in the late phase) and FHA (in the early phase). Stimulation with PRN peptide pools revealed lower levels of IL-13 in older adults in the early phase. Thus, despite various differences in study design and experimental approach between the SKI and Immfact samples, we observed a similar trend towards lower Th-type cytokine production in response to Bp antigens with increasing age.

### Similar Frequencies of Naïve and Memory Subsets and Regulatory Cells Within the CD4^+^ T-Cell Compartment of Youngsters and Older Adults Following Clinical Bp Infection

**FIGURE 4:**
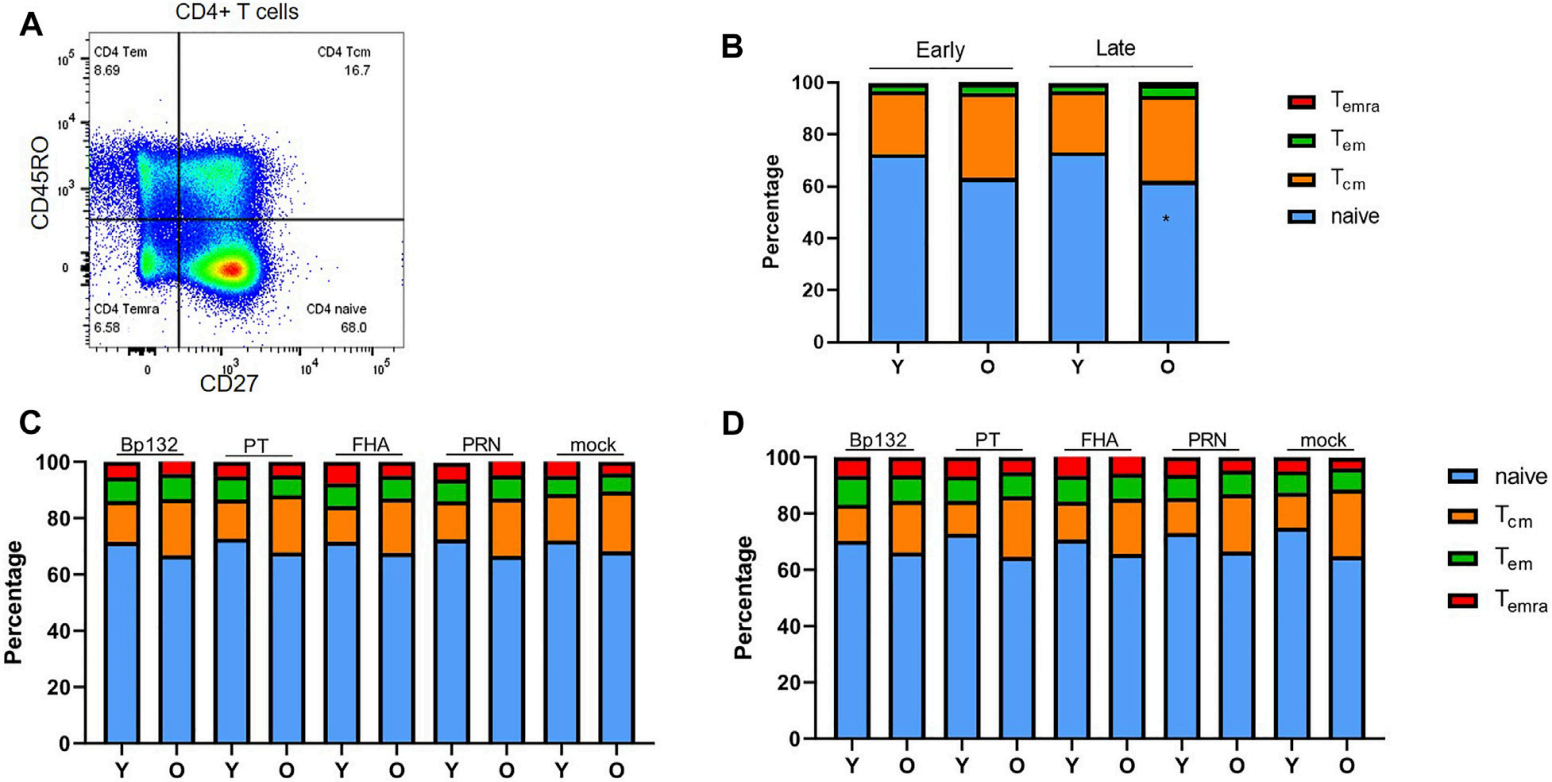
Frequencies of naïve and memory cells within the CD4^+^ T-cell population in longitudinal PBMC samples of youngsters (Y) and older adults (O) pre- and post *in vitro* culture. **(A)** Gating of naive and memory subsets based on CD45RO and CD27 expression. **(B)** Proportion of naïve/Tem/Tcm/Temra subsets within the CD4^+^ T-cell population at early and late phase prior to stimulation; **(C)** after 6-day antigenic stimulation in the early phase; and **(D)** after 6-day antigenic stimulation in the late phase. Stacked bars indicate median relative percentage of subsets per age group and timepoint. Statistical significance of proportional differences between CD4^+^ T-cell subsets of youngsters and older adults was calculated with Mann Whitney *U*-test. *p < 0.05.

We then explored CD4^+^ T-cell populations of the Immfact PBMC samples for the various flow-cytometric read-outs of our assay. To first assess whether youngsters and older adults of the Immfact cohort display differences in the frequencies of global naïve and memory CD4^+^ T-cell subsets in their peripheral blood, we analysed the CD4^+^ T-cell composition of PBMC samples prior to stimulation based on expression of CD27 and CD45RO (Figure 4A, gating strategy in Supplementary Figure S4). We found comparable percentages of central memory (T_cm_, CD45RO^+^CD27^+^), effector memory (T_em_, CD45RO^+^CD27^−^) and terminally differentiated (T_emra_, CD45RO^−^CD27^−^) subsets between the age groups at both early and late phase, only the percentage of naïve CD4^+^ T cells was significantly reduced in older adults compared to youngsters in the late phase (Figure 4B). After 6-day stimulation with Bp peptide pools Bp132, PT S1, FHA and PRN, we found the global composition of the CD4^+^ T-cell compartment to be comparable between youngsters and older adults and between early (Figure 4C) and late phase samples (Figure 4D).

Additionally, regulatory T cells (Tregs) have been suggested to accumulate with increasing age (Gregg et al., 2005; Lages et al., 2008; Elyahu et al., 2019). Therefore, we analysed the frequency of Tregs, defined as either CD127^-^CD25^+^ or FoxP3^+^CD25^+^ CD4^+^ T cells prior to stimulation (Seddiki et al., 2006; Yu et al., 2012; Rodríguez‐Perea et al., 2016). Tregs were in the range of 5–8% of the CD4^+^ T cells and frequencies were comparable between youngsters and older adults (Supplementary Figure S5. Altogether, these analyses show that youngsters and older adults from the Immfact cohort do not differ in the proportions of memory T-cell subsets (based on both phenotypes) before and after Bp-peptide stimulation, nor in *ex vivo* Tregs.

### Reduced Proliferative Responses of Bp-Specific CD4^+^ T Cells at Older Age

**FIGURE 5:**
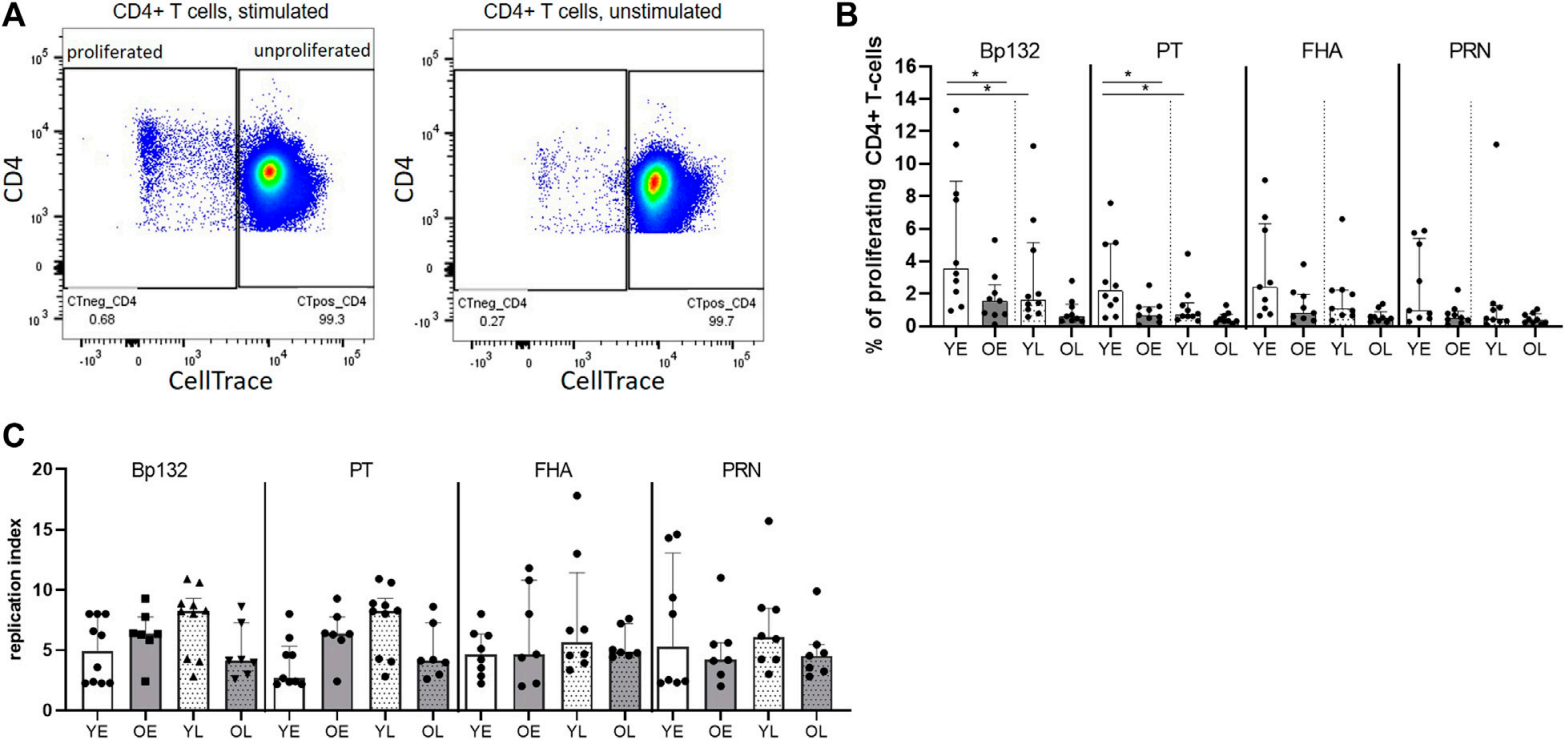
Reduced proliferative response of Bp-specific CD4^+^ T cells at older age. PBMCs of youngsters (Y) and older adults (O) of early (E) and late (L) timepoints were labelled with a proliferation dye and subsequently assessed on sequential halving of fluorescent intensity after 6-days stimulation Bp peptide pools Bp132, PT S1 (shown as PT), FHA, and PRN. **(A)** Gating strategy of proliferating and non-proliferating CD4^+^ T cells as shown for Bp132 peptide pool-stimulated (left plot) and medium (unstimulated) (right plot) conditions. **(B)** Frequencies of proliferating CD4^+^ T cells post stimulation. **(C)** Replication index. Dots show background subtracted frequencies of individual cases, while bars represent medians and interquartile range youngsters (Y), white bars; older adults (O), dark bars; early (E) phase samples, open bars; late (L) phase samples, dotted bars, as indicated. Statistical significance was calculated with Mann Whitney *U*-test and Wilcoxon matched pairs signed-rank test. * = *p* < 0.05.

Next, we investigated whether age had an impact on proliferative responses of antigen-specific CD4^+^ T cells in youngsters and older adults. To determine frequencies of proliferating CD4^+^ T cells, PBMCs were CellTrace labelled, stimulated with Bp antigen peptide pools for 6 days, and assessed for CellTrace Violet diminution (Figure 5A). Our data showed a general trend of lower frequencies of CD4^+^ T cells proliferating to Bp antigen in the older adults, which was significant for the Bp132 and PT S1 peptide pool early after clinical pertussis infection (Figure 5B). In the late phase, no age-related differences were found. We did observe waning of proliferative responses in youngsters in time, as frequencies of proliferated CD4^+^ T cells to Bp132 and PT S1 peptide pools were significantly lower in the late phase post infection compared to the early phase (Figure 5B). Patterns of Bp-specific CD4^+^ T-cell proliferation in older adults in response to FHA or PRN were comparable to those observed in youngsters. The proliferative response was further investigated by calculating the replication index (fold-expansion of proliferating cells). The replication index was comparable between youngsters and older adults (Figure 5C), suggesting that responding CD4^+^ T cells undergo equal rounds of cell divisions in adults compared to young individuals. Thus, these data show that the magnitude of the proliferative response of the CD4^+^ T-cell compartment to Bp antigens Bp132 and PT S1 declined with age but rounds of divisions in response to all Bp antigens per cell were similar. This suggests maintenance of a smaller memory pool of Bp-specific CD4^+^ T cells for recall responses as a hallmark of immunosenescence.

### Minor Phenotypical Differences of Proliferated CD4^+^ T cells Between Youngsters and Older Adults

Next, we assessed whether lower cytokine production and a lower proliferative response were accompanied by an altered phenotypical profile of responding CD4^+^ T cells. We hypothesized that antigen-specific, proliferating CD4^+^ T cells of older adults express higher levels of co-inhibitory receptors CTLA-4, PD-1, and TIGIT and lower levels of the activation marker CD25 (the IL-2-alpha receptor). In contrast, we found that expression of CTLA-4 by proliferated CD4^+^ T cells in the early phase in youngsters was significantly higher compared to older adults in response to Bp132, whereas the percentage of CTLA-4^+^ proliferating CD4^+^ T cells varied but was comparable between the two age groups (Supplementary Figures S6A,B). Expression of the activation marker CD25 on proliferated CD4^+^ T cells was comparable between youngsters and older adults both in the early and late phase, as well as were frequencies of CD25^+^ proliferated CD4^+^ T cells, both in the early and late phase (Supplementary Figures S6C,D). Likewise, analysis of expression and frequencies of PD-1^+^ (Supplementary Figure S7A–B) and TIGIT^+^ (Supplementary Figure S7C–D) cells within proliferating CD4^+^ T cells did not reveal age-related differences.

**FIGURE 6:**
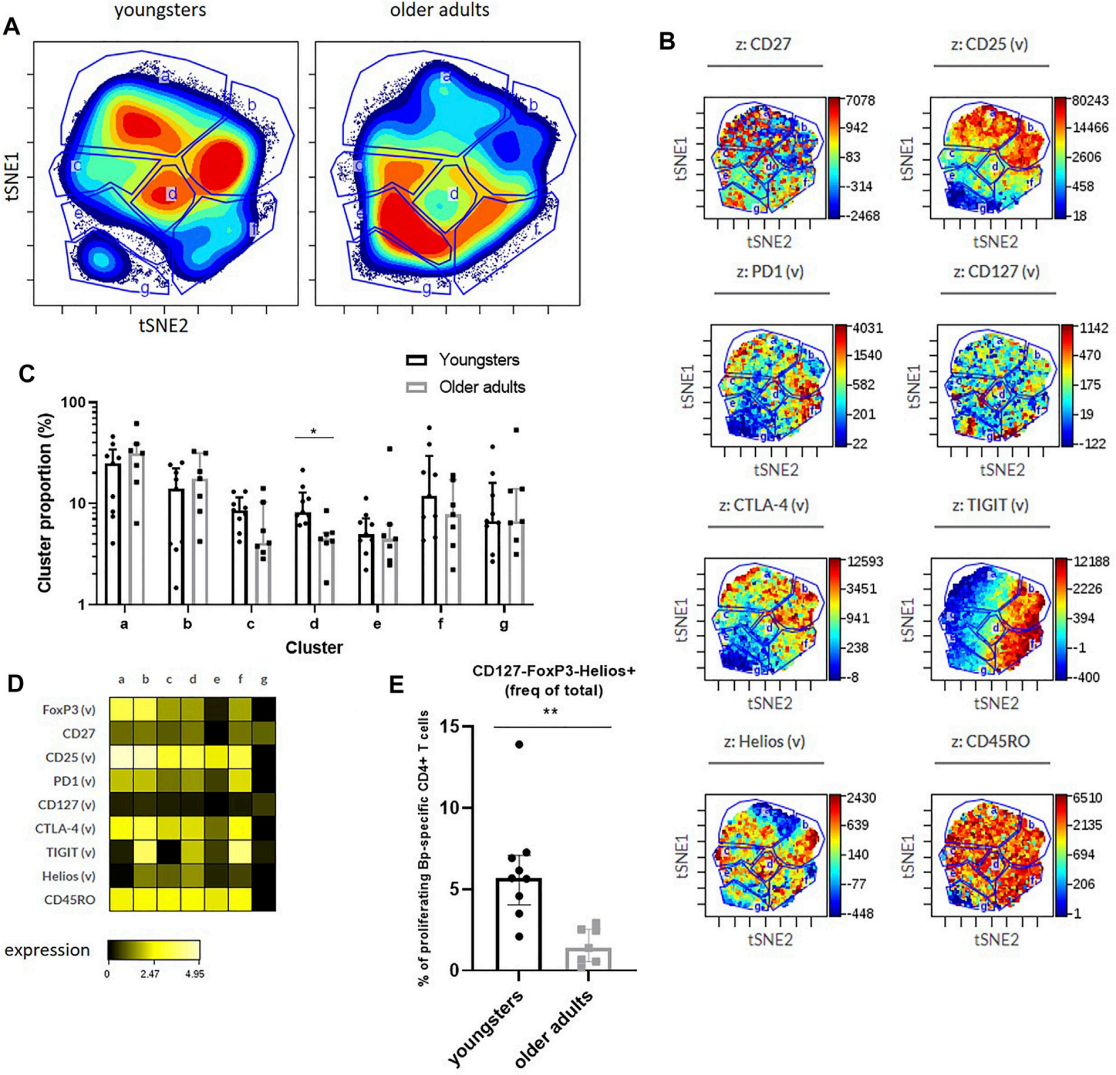
Minor age-related phenotypical differences of proliferated CD4^+^ T cells responding to Bp132 stimulation in youngsters and older adults in the early phase. **(A)** Cell density maps show phenotyping analysis by dimensionality reduced single cell data by viSNE show clustering of populations within the proliferating cells of youngsters and older adults of pooled flow cytometry datafiles pre-gated on proliferating CD4^+^ T cells and identified clusters indicated with letters a-g. **(B)** Dot-plot *z*-axis shows the location and intensities of each marker within the identified clusters. **(C)** Frequencies of identified viSNE clusters, based on their identifying marker expression as indicated, were compared on individual levels via FlowJo analysis. Bars represent median frequency of proliferated CD4^+^ T cells with interquartile range. Cluster proportions in youngsters and older adults as indicated identified by vISNE analyses. **(D)** Heatmap showing Arcsinh-transformed median expression of markers within each cluster of the viSNE plots of youngsters and older adults. **(E)** Frequencies of CD127^-^FoxP3^-^Helios^+^ proliferated CD4^+^ T cells in youngsters and older adults. Statistical significance was calculated with Mann Whitney *U*-test. * = *p* < 0.05, ** = *p* < 0.01.

Lastly, we applied dimensionality reduction analyses (viSNE) to proliferated CD4^+^ T cells of pooled datafiles of youngsters and older adults in the early phase to reveal any differences based on combined marker expression between proliferated CD4^+^ T cells of youngsters and older adults (Figures 6A,B). Cluster analysis showed a potentially interesting cluster, cluster d, that was significantly lower in older adults compared to youngsters (Figure 6C). Based on subsequent heatmap visualization of marker expression in the clusters (Figure 6D), this cluster was positive for markers including CD25, CTLA-4 and TIGIT and showed somewhat higher Helios expression compared to other clusters. Additionally, we applied the gating approach derived from cluster analysis of proliferated CD4^+^ T cells after pneumococcal antigen stimulation using a similar phenotypical marker panel (He et al., unpublished) and found significant higher proportion of CD4^+^ T cells that were CD127^-^FoxP3^-^Helios^+^ (Figure 6E). Helios is considered a marker associated with (CD4^+^) T-cell activation (Akimova et al., 2011; Bengsch et al., 2018). In summary, unbiased viSNE cluster analysis of Bp-specific CD4^+^ T cells that have proliferated did not reveal phenotypical differences between youngsters and older adults based on the expression of co-inhibitory receptors CTLA-4, PD-1, and TIGIT or of activation marker CD25. The significantly higher frequency of CD127^-^FoxP3^-^Helios^+^ proliferated CD4^+^ T cells in youngsters may however suggest a reduced activation status at older adult age. Altogether, these data indicate that older adult Bp cases show features of CD4^+^ T-cell Bp-specific immunosenescence following clinical infection, highlighted by significantly lower cytokine and proliferative responses than younger adolescent counterparts. Moreover, absence of age-related differences in fold-expansion and co-inhibitory marker expression of Bp-specific proliferated CD4^+^ T cells, with a significant reduction of an activated CD4^+^ T-cell subset at older age, suggest that the immunosenescence can largely be attributed to a reduced size and thereby responsiveness of the maintained Bp-specific memory CD4^+^ T-cell pool.

## Discussion

Bp-specific CD4^+^ T cells are key players in durable protection against Bp. In this study, we have shown for the first time that memory CD4^+^ T-cell responsiveness to Bp-specific antigens is impaired at older age. Our major findings are that both cytokine and proliferative responses of specific CD4^+^ T cells in the early contraction phase and the late maintenance phase after an *in vivo* recall by a clinical Bp infection were reduced in adult cases compared to younger cases who had a similar background of primary whole cell pertussis vaccination. These observations could either be explained by a reduced capacity to proliferate per CD4^+^ T cell, resulting in less cells to produce cytokines, or by maintenance of a reduced memory pool of Bp specific CD4^+^ T cells that could expand and produce cytokines in response to Bp, at older age. Evidence indicating a similar fold-expansion of proliferated CD4^+^ T cells of the age sub cohorts favoured the reduced memory pool hypothesis at older age, with the responding CD4^+^ T cells showing some hallmarks of reduced activation, but no co-inhibitory or regulatory features of immunosenescence.

IFNγ is essential in the clearance of pertussis infection (Barbic et al., 1997), potentiating bacterial opsonophagocytosis and killing by phagocytes (Lambert et al., 2019). Here, lower levels of IFNγ spots and corresponding concentrations in the supernatant to PT stimulation were found in adults compared to youngsters, possibly implicating reduced capacity to combat the Bp infection. Comparable IFNγ responses were found between age groups after FHA and PRN stimulation. This may be explained by differences in immunogenicity or *in vivo* expression of these antigens, or the fact that they are less unique for Bp than PT (Ashworth et al., 1982; Novotny et al., 1991). Extended analyses of cytokines in culture supernatants indicated that in the capacity of infected youngsters and older persons to produce a wide range of other Th cytokines, age again impacting the magnitude. Both in the early and late phase after clinical Bp infection, lower levels of produced cytokines, including IL-2, TNF-α, IL-5 and IL-13, were found with increasing age. The reduced cytokine levels with older age were found regardless of the type of stimulation or technical approach, and this was already apparent in the relatively younger adult participants from the SKI study and was confirmed in older adults of the Immfact study. Our approach and available clinical material in this study did not allow for intracellular cytokine analysis to identify cytokine producing cells. Although CD8^+^ T cells were shown by Dirix *et al.* to contribute to IFNγ responses to FHA stimulation, CD4^+^ T cells were regarded the main source (Dirix et al., 2012). In our study reduced IFNγ responses were also found against the Bp132 peptide pool, optimized for MHC class II restricted T-cell responses, and besides IFNγ other Th-type cytokine responses were found to be impacted by age. Therefore we regard CD4^+^ T cells as the main cell type responsible for the cytokine profiles observed. Further research is needed to elucidate mechanisms of immunosenescence of cytokine production or polyfunctionality at the single Bp-specific CD4^+^ T-cell level. Altogether, our data corroborate with a smaller maintenance and size of the Bp-specific CD4^+^ memory T-cell response, leading to reduced functionality at older age.

Reduced (CD4^+^) T-cell proliferative responsiveness is a hallmark of immunosenescence (Jiang et al., 2007). In the flow-based part of our study, we found lower frequencies of proliferated CD4^+^ T cells in response to *in vitro* Bp132 and PT S1 peptide pool stimulation in the early phase after clinical Bp infection at older age, but cells did not seem to differ in fold-expansion while proliferating, compared to their younger counterparts. Age-related limitations in levels of secreted IL-2, a cytokine that drives the proliferative response of activated T cells (Boyman and Sprent, 2012), could be a factor determining a smaller population of Bp-specific CD4^+^ T cells to clonally expand after activation. Our data were inconclusive with regards to this cytokine, since reduced IL-2 levels were measured in day-5 culture supernatants of PT-stimulated adult samples from the SKI-study, in contrast to Bp132 or PTS1-stimulated older adult samples from the Immfact study. CD25 expression on CD4^+^ T cells was heterogenous across youngsters and older adults, yet not significantly different. Hence, the data do not indicate reduced availability of the IL-2 receptor alpha chain. As a mechanism for reduced CD4^+^ T-cell responsiveness, accumulation of Tregs, main cells expressing high levels of CD25 and consuming IL-2 in human peripheral blood, at older age has been put forward (Gregg et al., 2005; Lages et al., 2008; van der Geest et al., 2014), although some studies find unaltered (Valmori et al., 2005; Hwang et al., 2009) frequencies of Tregs with higher age. In our study we did not observe an increased frequency of Tregs after clinical infection in older adults compared to youngsters, not favouring of a major role for Tregs in IL-2 consumption or reduced CD4^+^ memory T-cell proliferation in older adults in our study.

Using hypothesis-driven and unbiased viSNE cluster analysis of the flow-cytometric dataset we explored the hypothesis that enhanced expression of co-inhibitory receptors CTLA-4, PD-1, and/or TIGIT on responding CD4^+^ T cells at older age was associated with reduced Bp-specific CD4^+^ T-cell responsiveness, as reported to explain impaired T-cell proliferation within the T-cell population at older age in mice and humans (Leng et al., 2002; Lages et al., 2010; Bengsch et al., 2018; Song et al., 2018). In contrast to our hypothesis, we found that phenotypical markers for T-cell activation and co-inhibition did not significantly differ between youngsters and older adults, instead proliferated Bp-specific CD4^+^ T cells of youngsters had a higher MFI of CTLA-4 expression in response to Bp132, possibly in response to stronger activation. Notably, the proliferated population of youngsters also contained of a significantly higher frequency of CD127^-^FoxP3^-^Helios^+^ cells, considered to represent (CD4^+^) T-cell activation (Gregg et al., 2005; Lages et al., 2008). Thus, our data seem to indicate that immunosenescence observed in the proliferative responsiveness of Bp-specific CD4^+^ T cells at older age is not associated with increased hallmarks of negative regulation but rather with decreased phenotypical markers that are associated with activation.

Our data suggest that immunosenescence in the Bp-specific CD4^+^ T-cell response may occur already in relatively young adults. Likewise, in a cohort of 18–49 year old individuals, influenza-specific T-cell responses were shown to become less diverse and less cross-reactive with age (Subbramanian et al., 2010), a trend which extended into shorter-lived booster T-cell responses in a cohort of older adults (≥70 years old) vaccinees (Mahnke et al., 2011). We earlier documented reduced in Bp-specific proliferative responsiveness in ex-pertussis cases, using a 3H thymidine assay and whole PT or single PT and PRN CD4^+^ T-cell epitopes as antigenic stimulation, starting at the age of 30 years (Han et al., 2013; van Twillert et al., 2015). Together our data now suggest that decline of responsiveness may reflect a gradual loss in the diversity of epitopes recognized.

Adults in these studies may have encountered *B. pertussis* antigens more frequently through natural exposure, considering the existing circulation. Repeated encounters with *B. pertussis* could possibly lead to end-stage differentiation of memory CD4^+^ T cells with a CCR7-CD27^−^phenotype, associated with shorter telomeres and less proliferative capacity than early-stage CCR7^+^CD27^+^ memory CD4^+^ T cells (Fritsch et al., 2005). Also, inherent to the study design, in adults Bp-specific CD4^+^ memory T cells primed through childhood vaccination experienced a substantially longer maintenance period preceding the clinical pertussis event, studied in this work, compared to their counterparts in the younger group. Together, these factors may confound effects of true immunosenescence, a limitation of these kind of clinical pathogen-specific and age-related clinical cohort studies.

Another limitation of our study is that the early phase clinical samples in the SKI and Immfact studies were taken only at a time point between 0.5 and 3 months post-diagnosis, respectively, likely missing peak levels reached during the expansion phase of specific CD4^+^ memory T-cell subsets that could occur within days after infection. Instead, levels of responding cells represent the contraction phase as they are still elevated and further declining to long-term maintenance levels. In addition, sampling within the SKI study was cross-sectional and was variable with regards to median time elapsed since diagnosis between younger and older age cohorts, as opposed to Immfact sampling which was longitudinal and homogeneous in time frames between age cohorts. It is however unlikely that enhanced late phase cytokine production in younger SKI samples could be attributed to differential sampling times, as the median late point sampling time of youngsters in the SKI study was longer after diagnosis compared to the adults. Additionally, the type of pertussis vaccine for primary vaccination, whole cell or acellular vaccine, is known to have a long-lasting impact on important hallmarks of subsequent Bp-specific CD4^+^ T-cell immunity in response to recall (reviewed in (Ausiello et al., 2019), recently confirmed by (da Silva Antunes et al., 2021)). Primary vaccination for all patients in our study was with whole cell vaccine, however several Immfact youngsters received an additional aP booster dose at pre-school age. These subjects did not cluster for their cytokine or proliferative responses, but we cannot fully exclude the influence of this aP booster vaccination. Lastly, the observational natural infection studies SKI and Immfact provide unique cohorts of subjects to study hallmarks of Bp-specific CD4^+^ T-cell immunity after recovery from a clinical infection. However, the number of ex-patients eligible for the selected age strata of this work was small in both clinical cohorts. Small group size may have limited the power to find age effects in our data. Further in depth research at the single Bp-specific CD4^+^ cell level on additional samples are needed to confirm and extent these findings.

In addition to epidemiological studies, serosurveillance studies have highlighted (symptomatic) infections in the elderly as well (Hodder et al., 2000; de Greeff et al., 2010). High-dimensional evaluation of antibody levels following a Bp infection at older age compared to younger age indicates that antibody specificity, isotype and subclass of antibodies are all impacted by age and primary vaccination background (van Twillert et al., 2017). No correlate of protection has been established for pertussis, yet. The fact that pertussis occurs in all age groups, with underreporting in elderly, highlights the importance of further understanding the Bp-specific immune responses and changes therein with progressing age. As a possible intervention to enforce pertussis specific immunity, it was recently shown that older adults do mount both antibody and CD4^+^ T-cell booster responses to an acellular pertussis booster dose (Lambert et al., 2020; Versteegen et al., 2021). Our study here, however, for the first time supports the hypothesis that Bp-specific memory CD4^+^ T-cell immunity is subject to aging, possibly contributing to a more rapid loss of protective immune mechanisms with increasing age. For a more effective control of pertussis, also in the growing elderly population, induction and maintenance of protective CD4^+^ T-cell responses to Bp should be better understood.

## Data Availability

The raw data supporting the conclusions of this article will be made available by the authors, without undue reservation.
