# Supplementary material for: Reduced Bordetella pertussis-specific CD4+ T-Cell Responses at Older Age
Source: Front Aging. 2022 Feb 2;2:737870. doi: 10.3389/fragi.2021.737870 (PMC9261443; doi:10.3389/fragi.2021.737870)
Supplement: Supplementary file 1 [file DataSheet1.docx]

Supplementary Material

To

Reduced *Bordetella pertussis*-specific CD4+ T-cell responses at older age

Eleonora. E. Lambert, Inonge van Twillert, Lisa Beckers, Martien C.M. Poelen, Wanda G. H. Han, Daan K. J. Pieren, and Cécile A.C.M. van Els

**
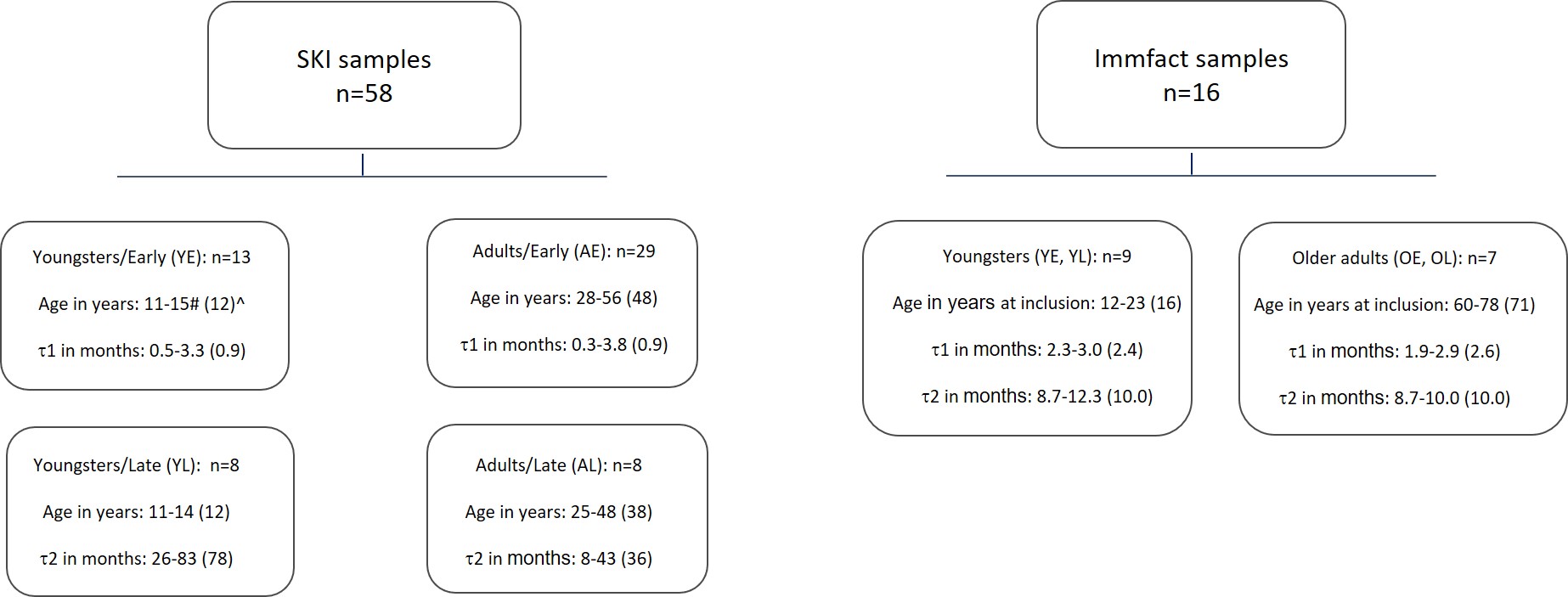
**

**Supplementary Figure 1. Flowchart of study populations.** Subjects are stratified by clinical study and by age subgroup: youngsters (Y), adults (A) or older adults (O). Indicated per subgroup are number (n) of subjects, age and time (τ) of sampling. # = range; ^median; τ1 = early (E) time point in months since diagnosis of clinical infection; τ2 late (L) time point in months since diagnosis of clinical infection

**
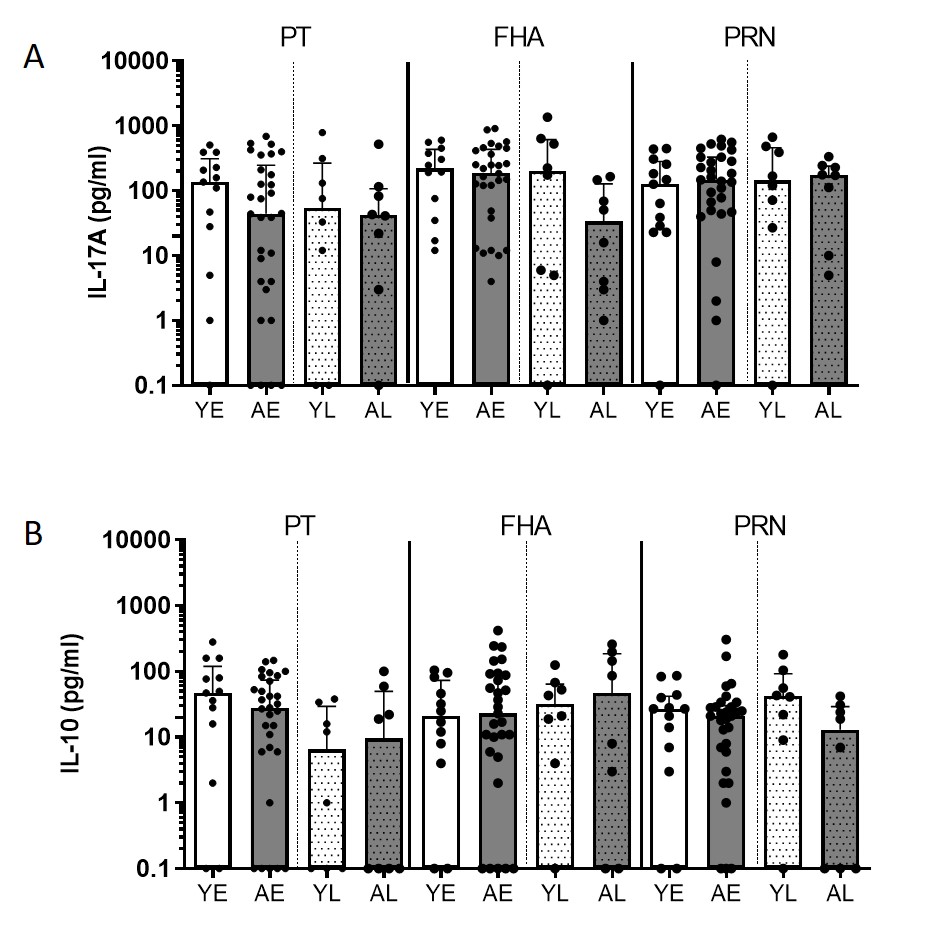
**

**Supplementary Figure 2. Supernatant-based analysis of Bp-specific IL-17A and IL-10 responses in youngsters and adults in early and late phase after clinical infection (SKI). (A)** IL-17A and **(B)** IL-10 in supernatants of PBMCs *in vitro* stimulated with Bp protein stimulations PT (left panels), FHA (middle panels) and PRN (right panels) in youngsters (white bars) and adults (dark bars) in early (open bars) and late (dotted bars) phase after clinical diagnosis (cross sectional study). Dots show individual cases while bars indicate medians and interquartile range. Statistical significance was calculated with Mann Whitney *U*-test.

**
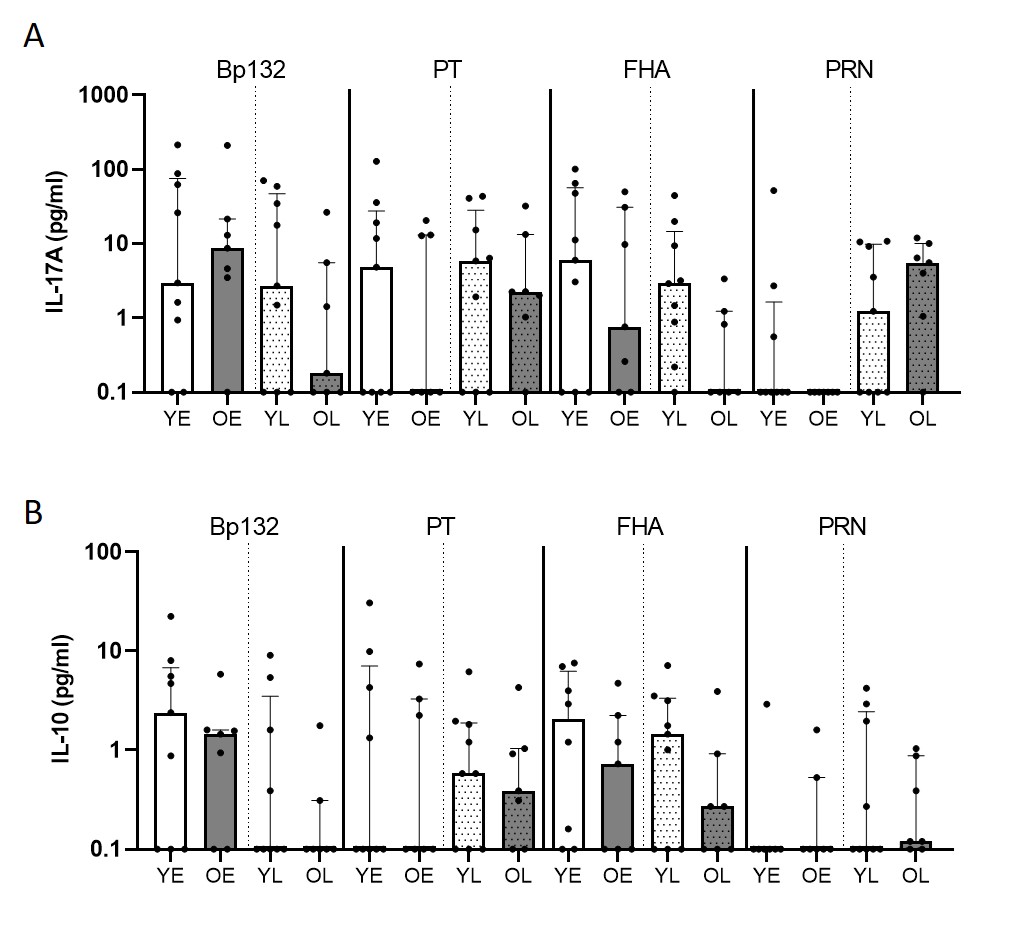
**

**Supplementary Figure 3. Supernatant-based analysis of Bp-specific IL-17A and IL-10 responses in youngsters and older adults in early and late phase after clinical infection (Immfact). (A)** IL-17A and **(B)** IL-10 in supernatants of PBMCs *in vitro* stimulated with peptide pools Bp132, PTX S1 (shown as PT), FHA and PRN, as indicated, in youngsters (Y, white bars) and older adults (O, dark bars) in early (E, open bars) and late (L, dotted bars) phase after clinical diagnosis (longitudinal clinical study). Dots show individual cases while bars indicate medians. Statistical significance was calculated with Mann Whitney *U*-test.


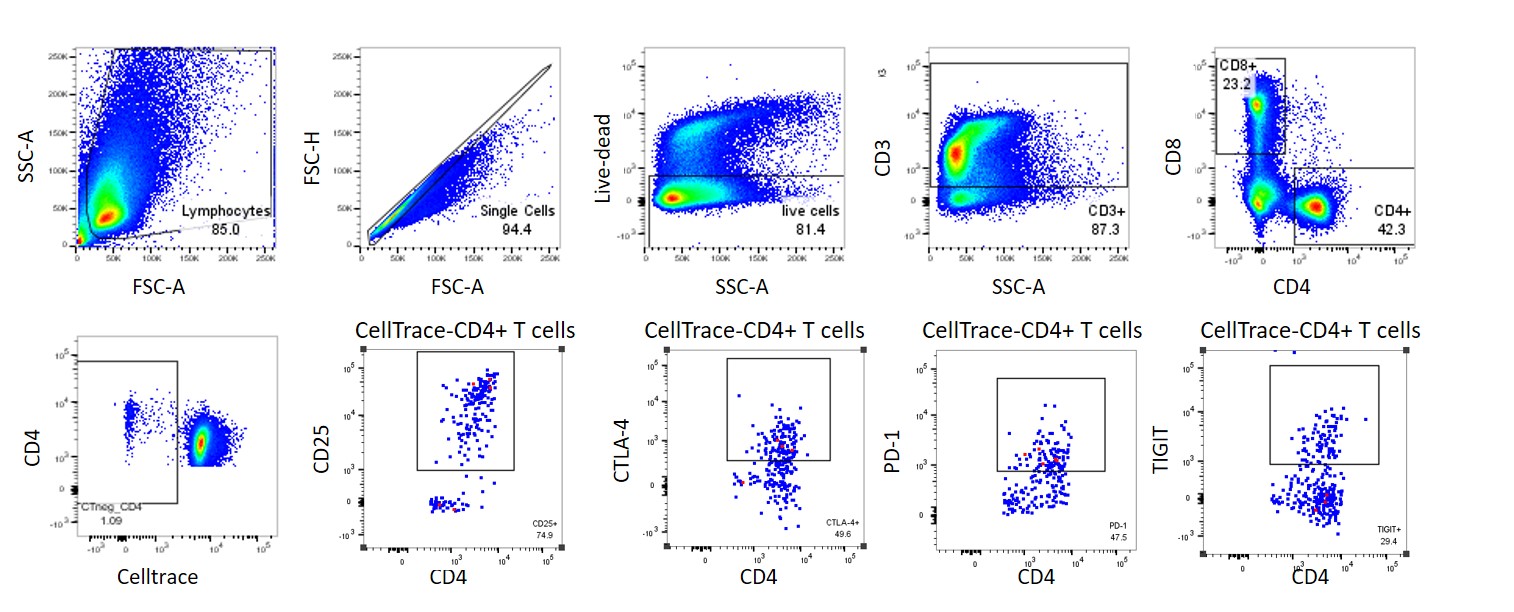


**Supplementary Figure 4. Flow cytometry gating strategy for CD4+ T-cell populations.** Sequential steps in gating of lymphocytes, singlets, live cells, CD3+ cells, and CD4+ T cells, as indicated in left to right plots in the upper panel, and of proliferated CellTrace-CD4+ T cells in the left plot in the lower panel, respectively; gating strategy to determine MFI and frequencies of selected markers within proliferated CellTrace-CD4+ T cells in Bp-antigen stimulation conditions, as indicated in the four right plots in the lower panel.

**
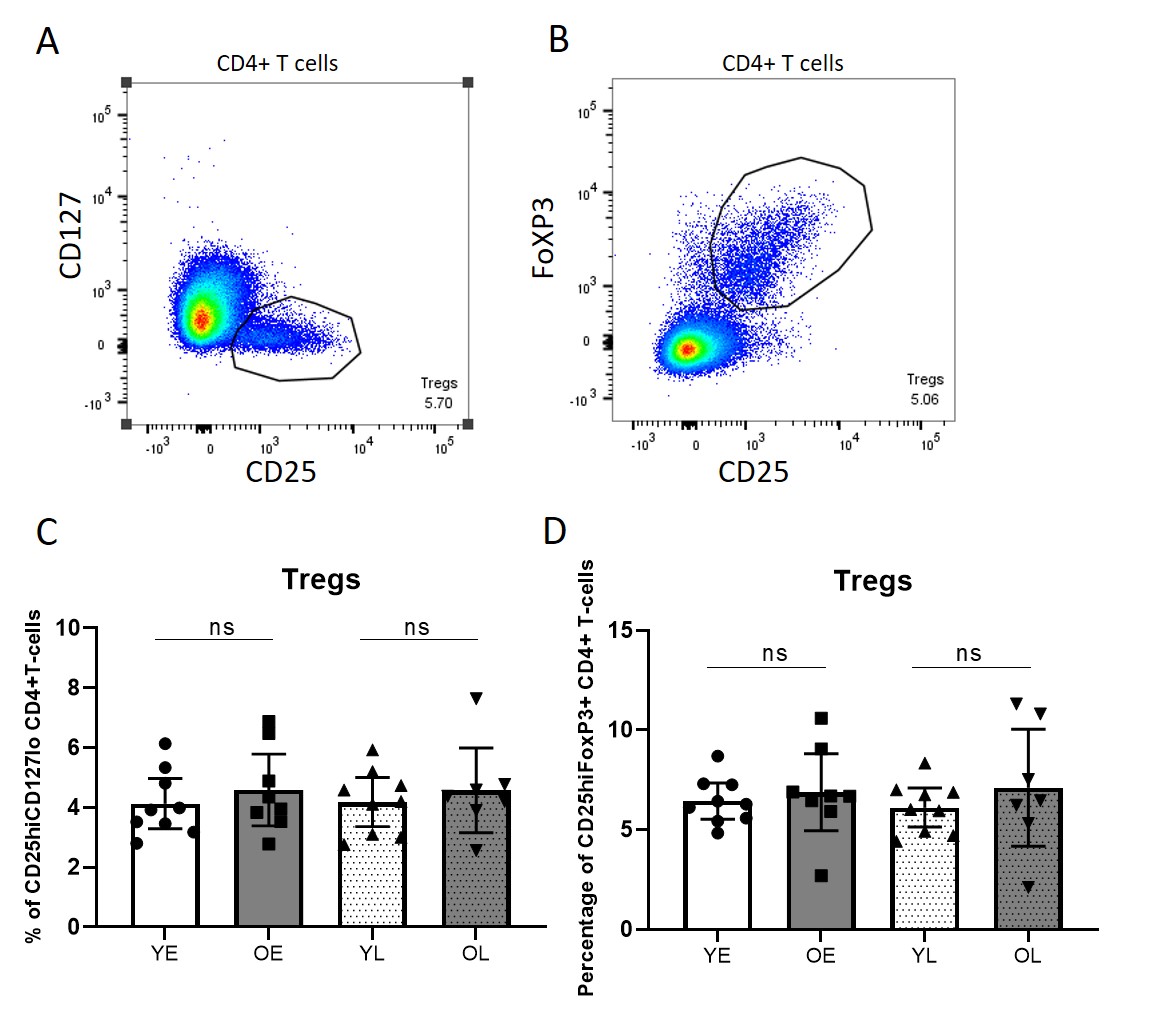
**

**Supplementary Figure 5. Similar frequencies of CD4+ Tregs between youngsters and older adults.** PBMCs of youngsters and older adults in early and late phase were ex vivo assessed for their frequencies of CD4+ Tregs. Gating of CD4+ Tregs based on **(A)** expression of CD25 and CD127; and of **(B)** CD25 and FoxP3, as indicated by gates, respectively; **(C)** Frequency of CD4+ Tregs based on expression of CD25 and CD127; and **(D)** of CD25 and FoxP3. Bars indicate medians with interquartile range of youngsters (‘Y’, white bars) and older adults (‘O’, grey bars) within 3 months (early ‘E’, open bars), or 9-12 months (late ‘L’, dotted bars) after diagnosis. Statistical significance was calculated with Mann Whitney *U*-test, ns=not significant.


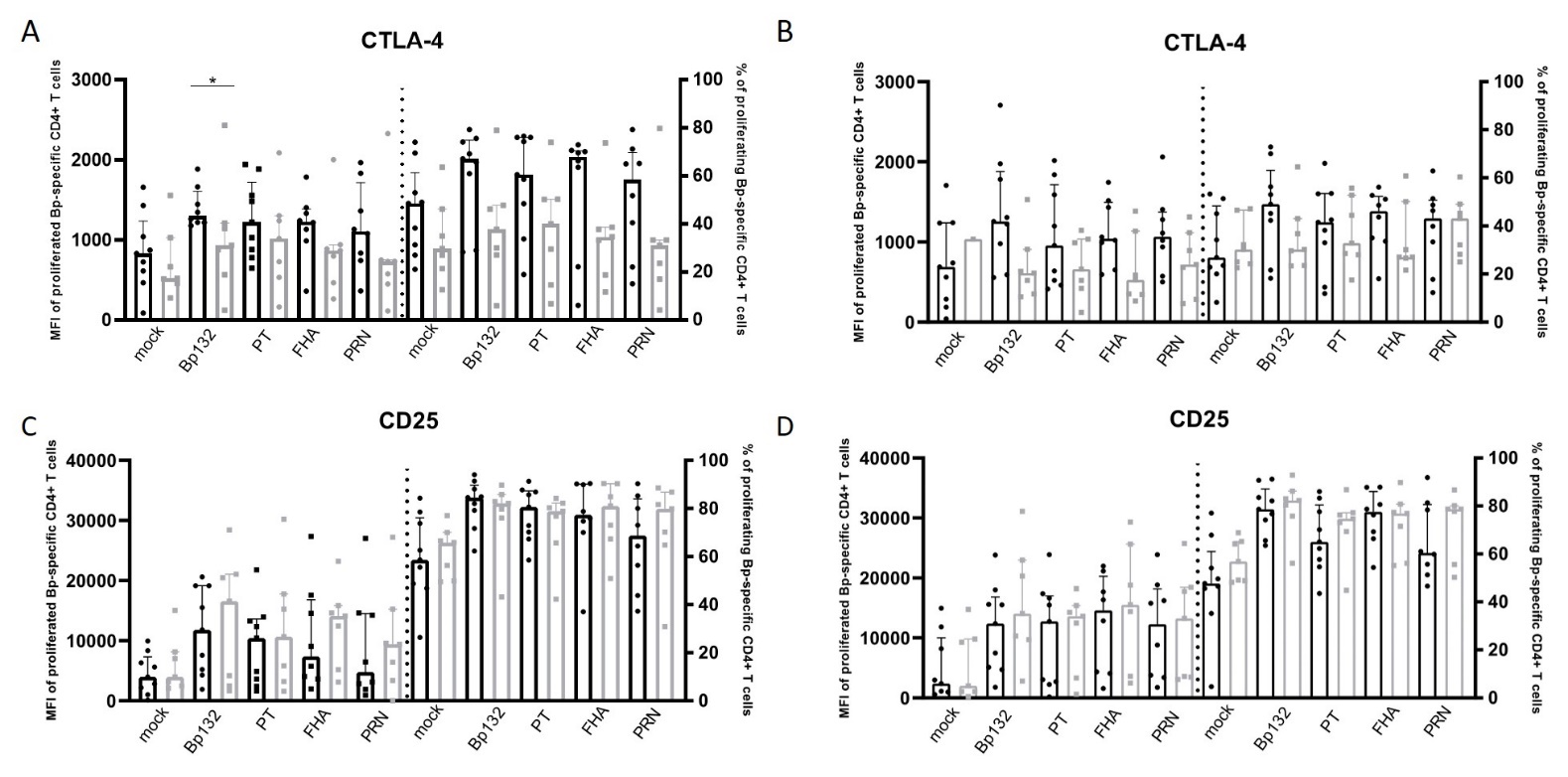


**Supplementary Figure 6. Minor phenotypical differences of proliferating CD4+ T cells between youngsters and older adults.** PBMCs of youngsters (black bars) and older adults (grey bars) were stimulated for 6 days with the Bp132 peptide pool or with peptide pools of PT S1 (shown as PT), FHA, and PRN, or mock, as indicated. **(A)** MFI (bars left of the dotted line) and percentage (bars right of the dotted line) of CTLA-4 in proliferated Bp-specific CD4+ T cells, in the early phase after clinical pertussis diagnosis. **(B)** MFI and percentage of CTLA-4 in proliferated Bp-specific CD4+ T cells, in the late phase after clinical pertussis diagnosis. **(C)** MFI and percentage of CD25 in proliferated Bp-specific CD4+ T cells, in the early phase after clinical pertussis diagnosis. **(D)** MFI and percentage of CD25 in proliferated Bp-specific CD4+ T cells, in the late phase after clinical pertussis diagnosis. Dots show individual cases, bars represent medians with interquartile range. Statistical significance was calculated with Mann Whitney *U*-test. *= *p* <0.05

**
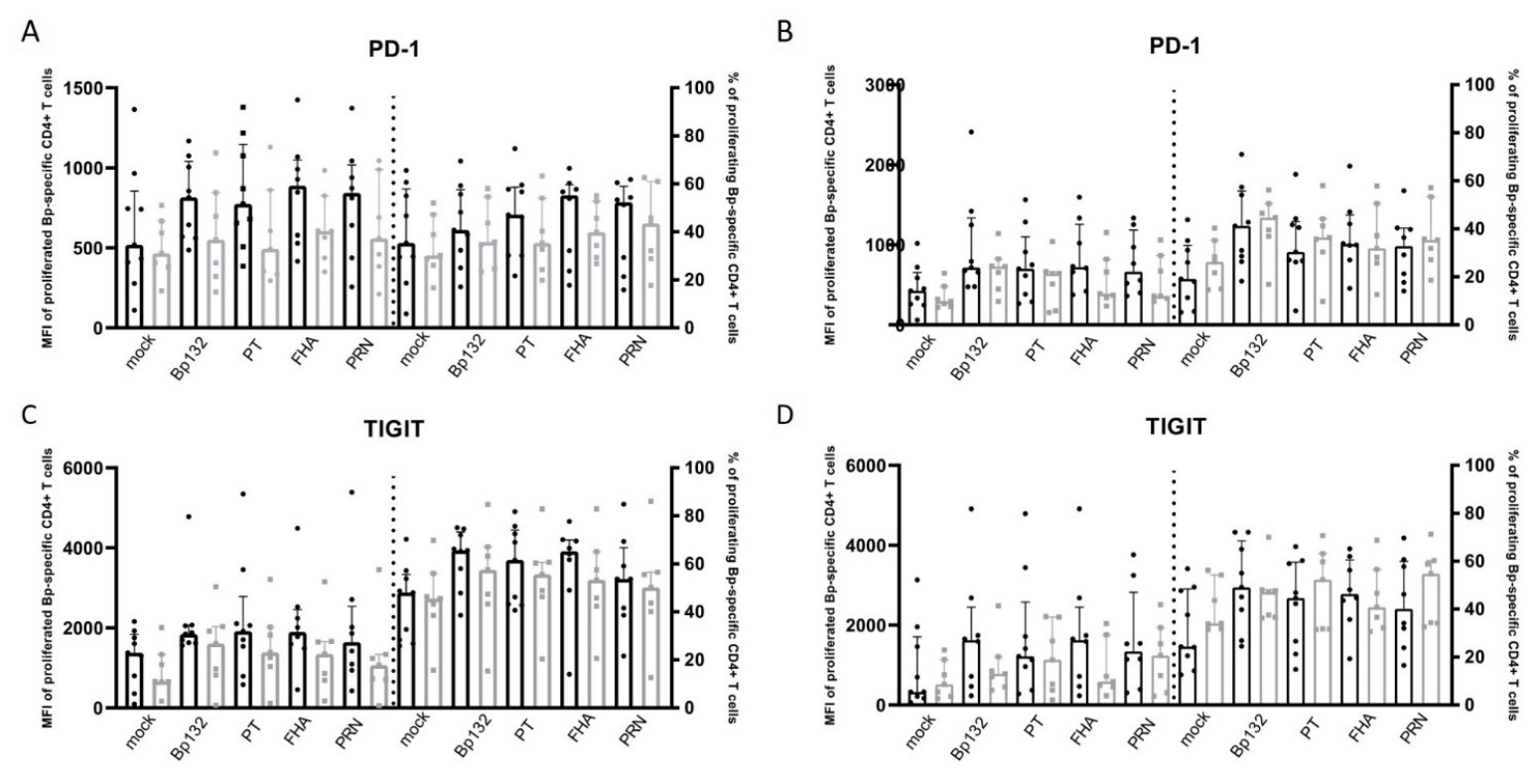
**

**Supplementary Figure 7. No age-related differences in PD-1 or TIGIT expression of proliferating CD4+ T cells in youngsters and older adults.** PBMCs of youngsters (black bars) and older adults (grey bars) were stimulated for 6 days with the Bp132 peptide pool or with peptide pools of PT S1 (shown as PT), FHA, and PRN, or mock, as indicated. **(A)** MFI (bars left of the dotted line) and percentage (bars right of the dotted line) of PD-1 in proliferated Bp-specific CD4+ T cells, in the early phase after clinical pertussis diagnosis. **(B)** MFI and percentage of PD-1 in proliferated Bp-specific CD4+ T cells, in the late phase after clinical pertussis diagnosis. **(C)** MFI and percentage of TIGIT in proliferated Bp-specific CD4+ T cells, in the early phase after clinical pertussis diagnosis. **(D)** MFI and percentage of TIGIT in proliferated Bp-specific CD4+ T cells, in the late phase after clinical pertussis diagnosis. Dots show individual cases, bars represent medians with interquartile range. Statistical significance was calculated with Mann Whitney *U*-test.
